# Lyssaviruses and Bats: Emergence and Zoonotic Threat 

**DOI:** 10.3390/v6082974

**Published:** 2014-08-04

**Authors:** Ashley C. Banyard, Jennifer S. Evans, Ting Rong Luo, Anthony R. Fooks

**Affiliations:** 1 Wildlife Zoonoses and Vector Borne Disease Research Group, Animal Health and Veterinary Laboratories Agency, Weybridge, New Haw, Surrey KT15 3NB, UK; E-Mails: jenni.evans@ahvla.gsi.gov.uk (J.S.E.); tony.fooks@ahvla.gsi.gov.uk (A.R.F.); 2 University of Warwick, Gibbet Hill Road, Coventry, West Midlands CV4 7AL, UK; 3 State Key Laboratory for Conservation and Utilization of Subtropical Agro-Bio-Resources, College of Animal Sciences and Veterinary Medicine, Guangxi University, Nanning 530004, China; E-Mail: tingrongluo@gxu.edu.cn; 4 Department of Clinical Infections, University of Liverpool, Microbiology and Immunology, Daulby Street, Liverpool L69 3GA, UK

**Keywords:** rabies, bats, lyssaviruses, emergence, zoonoses

## Abstract

The continued detection of zoonotic viral infections in bats has led to the microbial fauna of these mammals being studied at a greater level than ever before. Whilst numerous pathogens have been discovered in bat species, infection with lyssaviruses is of particular significance from a zoonotic perspective as, where human infection has been reported, it is invariably fatal. Here we review the detection of lyssaviruses within different bat species and overview what is understood regarding their maintenance and transmission following both experimental and natural infection. We discuss the relevance of these pathogens as zoonotic agents and the threat of newly discovered viruses to human populations.

## 1. Introduction

**Figure 1:**
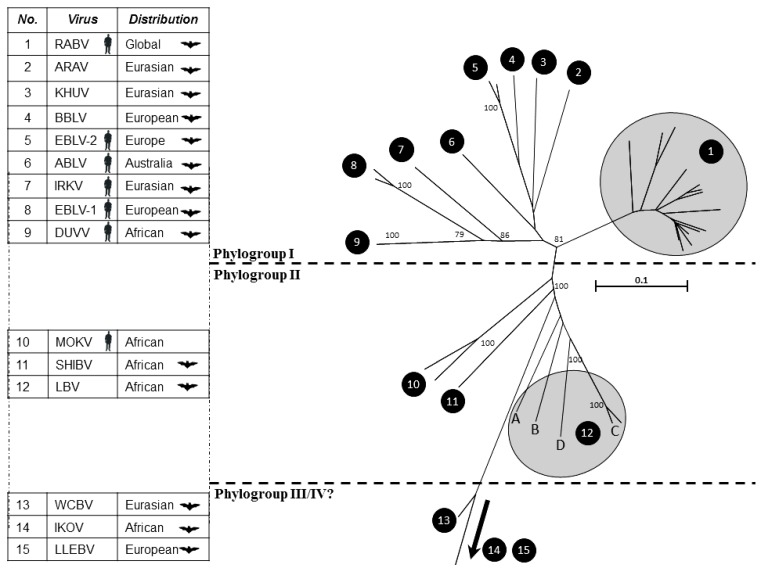
A phylogenetic analysis of the lyssaviruses. Numbers on the tree correspond to each of the lyssaviruses detailed in the accompanying table. Silhouetted species represent where viruses have been associated with bat infection and/or human fatalities. Virus acronyms are as defined in Table 1. The phylogenetic analysis is based on 405 nucleotides of the nucleoprotein gene. All sequences were aligned using ClustalW. Bootstrap values at significant nodes are shown.

Rabies virus (RABV) is one of the oldest and most feared viruses in the history of mankind. Its association with bats is the basis for many mythical legends and imagery and the virus plays an important role in the human perception and general fear of bats. Whilst in the case of blood sucking hematophagous bats, some of this inherent fear may be justified, the vast majority of bat species worldwide pose little threat to human populations. Rabies virus is a lyssavirus and is just one virus species in an ever expanding genus termed the lyssaviruses [1]. Importantly, as with rabies virus, the majority of the other lyssaviruses that make up this group of 14 distinct viral species, have been detected in bats (Table 1) and bats are considered to represent the ancestral host for this group of viruses [2,3]. Only two lyssaviruses, namely Mokola virus (MOKV) and Ikoma Lyssavirus (IKOV), have not been directly implicated in infections of bats, though evolutionary analyses and assessment of virus–host relationships suggest that all lyssaviruses, including RABV, most likely originated in bats [2,3]. The genetic relationship between the lyssaviruses and, where reported, association of lyssavirus infection with bat species is depicted in Figure 1. The viruses that have caused human fatalities are also indicated within the figure.

**Table 1:** Association of lyssaviruses with different bat species. * only genetic data has been reported for LLEBV.

Geographical distribution	Lyssavirus species	Bat species most commonly associated with lyssavirus infection	Common name	Transmission from bats implicated in human fatalities
The Americas	Rabies virus (RABV)	*Eptesicus fuscus*	Big brown bat	Yes
		*Tadarida brasiliensis*	Mexican/Brazilian free-tail bat	Yes
		*Lasionycteris noctivagens*	Silver-haired bat	Yes
		*Perimyotis subflavus*	Tri-coloured bat	Yes
		*Desmodus rotundus*	Vampire bat	Yes
Africa	Lagos Bat Virus (LBV)	*Eidolon helvum*	Straw coluored fruit bat	No
		*Rousettus aegyptiacus*	Egyptian fruit bat	No
		*Epomorphorus wahlbergi*	Wahlberg’s epauletted fruit bat	No
	Shimoni Bat Virus (SHIBV)	*Hipposideros commersoni*	Commerson’s leaf-nosed bat	No
	Duvenhage virus (DUVV)	*Miniopterus sp?*	Undefined	Yes
		*Nycteris thebaica*	Egyptian slit-faced bat	Yes
Eurasia	European Bat Lyssavirus type 1 (EBLV-1)	*Eptesicus serotinus*	Serotine bat	Yes
	European Bat Lyssavirus type 2 (EBLV-2)	*Myotis daubentonii*	Daubenton’s bat	Yes
	Bokeloh Bat Lyssavirus (BBLV)	*Myotis nattereri*	Natterer’s bat	No
	Aravan virus (ARAV)	*Myotis blythi*	Lesser mouse-eared bat	No
	Irkut Virus (IRKV)	*Murina leucogaster*	Greater tube-nosed bat	Yes
	Khujand Virus (KHUV)	*Myotis mystacinus*	Whiskered bat	No
	West Caucasian Bat Virus (WCBV)	*Miniopterus schreibersii*	Common bent-winged bat	No
	Lleida Bat Lyssavirus (LLEBV) *	*Miniopterus schreibersii*	Common bent-winged bat	No
Australasia	Australian Bat Lyssavirus (ABLV)	*Pteropus alecto*	Black flying fox and related sp.	Yes
		*Saccolaimus flaviventris*	Yellow-bellied sheath-tailed bat	Yes

**Figure 2:**
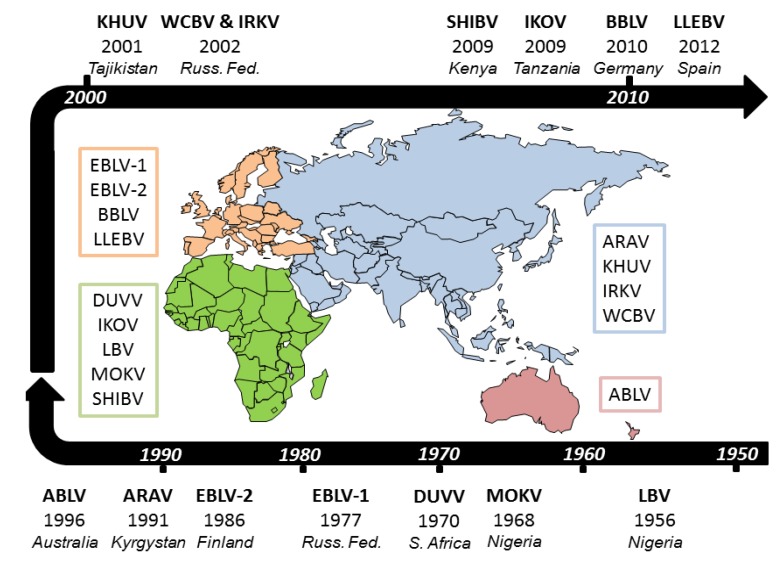
A Lyssavirus timeline. Acronyms are as detailed in Table 1. Regions where different lyssaviruses are found are coloured and dates for initial isolations are shown.

Until the 1950s, the association of rabies virus with bats was restricted to the situation understood in the Americas where both Vampire bat rabies and rabies of insectivorous bats had been described [4,5]. Initial detections of rabies in insectivorous bats was thought most likely to be due to transmission from Vampire bats and it was not until much later that a role for insectivorous bats became evident in North America [6]. From the 1950s, with the development of antibody typing methods and later molecular techniques to characterise viruses, it became clear that bats in the Old World harboured a number of viruses that were genetically and antigenically related to rabies virus but were different enough to be considered virus types in their own right. Historically, the discovery of novel lyssaviruses started with the detection of a viral agent named Lagos Bat Virus (LBV), in the pooled brains of Straw coloured fruit bats (*Eidolon helvum*), at Lagos Island, Nigeria [7]. At that time, due to the absence of Negri bodies, then a common diagnostic marker for rabies virus, and an inability of rabies virus specific serum to neutralise the novel virus, an association of LBV with rabies was not made. In Europe a handful of cases of bat rabies were also being reported [8] alongside the detection of rabies in insectivorous bats in North America [6]. A further virus, Mokola virus, was then discovered in extra neural tissues of shrews in Nigeria and in the following years from numerous mammalian species, including humans, across Africa [9,10,11,12,13,14,15,16]. A further novel virus, Duvenhage virus (DUVV) was isolated following a human case of rabies like disease in South Africa [17] and detection of DUVV in insectivorous bats followed [18]. By 1985 several European bat rabies cases had been reported and with the infection of a human in Finland in 1986 with European Bat lyssavirus type-2 (EBLV-2), novel lyssaviruses had been implicated in human fatalities. The increased interest in bats, through the discovery of lyssaviruses that constitute a threat to both human and animal health, alongside other viruses that have been discovered in bats has led to the study of this Order of mammals on an unprecedented scale [19,20,21,22,23]. Due to this increased interest in bat species and the pathogens they harbour, over the last decade a further seven lyssaviruses have been reported, the majority in bat species. A timeline overviewing the initial detection of each of the lyssaviruses and their predicted global distribution can be seen in Figure 2.

A remarkable conundrum exists regarding the detection of lyssaviruses across the globe. In the Americas, only RABV has been detected in both bats and terrestrial carnivore species, the latter constituting the most significant threat to human health posed by these viruses across this continent. Interestingly, no other lyssaviruses have been reported across the New World. In contrast, across the Old World, Europe, Africa, Eurasia and Australasia, whilst RABV exists in terrestrial hosts, there appears to be a complete lack of RABV in chiropteran species. Instead, other lyssaviruses are found sporadically across the Old World. A basis for this apparent restriction in virus distribution remains unclear, and with the inability of the most commonly used antigen based diagnostic assays to differentiate between lyssavirus species, the epidemiological significance is uncertain. 

## 2. Infection of Different Bat Species

The association of different lyssaviruses with different bat species has been reviewed extensively [24]. As mentioned, the reason behind the apparent exclusion of viruses from different geographical regions is unclear. Defining host restriction through either viral or host genetics is problematic for this group of viruses as, for the majority of the lyssaviruses, very few isolates exist. Where identified, the bat species most commonly associated with detection of each of the lyssaviruses are tabulated in Table 1. Clearly, whilst some lyssaviruses appear able to infect a diverse range of bat species, others appear restricted to certain species of bat. Certainly, for RABV, virus or virus neutralising antibodies have been detected in numerous bat species [25,26,27,28,29,30,31,32]. In contrast where human infection is reported, a comparatively small number of different bat species are most likely involved [30]. For the lyssaviruses of the Old World, the comparatively low frequency of detection makes a thorough assessment difficult. One exception to this is seen with European bat lyssaviruses where, whilst occasionally associated with other species, EBLV-1 is most commonly associated with the Serotine bat whilst EBLV-2 infection is most commonly associated with the Daubenton’s bat [33]. The biological bases for the perceived virus-host restrictions remain completely unknown.

## 3. Transmission and Maintenance

The mechanisms by which bats maintain lyssaviruses within roosts is currently undefined. This is most profoundly affected by the paucity of knowledge surrounding bat ecology and epidemiology. Whilst lyssaviruses have been detected as infecting a wide range of bat species across the globe, the diversity in their habitation, life cycles and the variable population sizes of different species may all affect the maintenance of lyssaviruses. Alongside this, some bat species appear restricted in their location whilst others have extensive geographical distributions that span continents. The basis for this fundamental difference in habitation and migratory tendencies remains unknown. What is clear is that although some bat species show a predilection for certain roost sites, other species can disperse to unknown locations travelling great distances between perceived roost sites [34]. This latter feature is particularly evident for the large frugivorous bats of Africa that have been shown to migrate several thousand kilometres, moving as far as 370 km in a single night flight. Drivers for this movement are not understood although recent studies have assessed population genetics for one species of large migratory African fruit bat and have suggested that such movements represent a significant factor in the dissemination of zoonotic viruses [35]. It is hoped that an increased understanding of the structures of migratory fruit bat metapopulations and the way in which populations interact across continents may enhance our understanding of lyssavirus maintenance.

In the case of both insectivorous bat populations across the Americas and the migratory African Fruit bats, the high roost densities reported for some bat species may facilitate a mode of virus maintenance within populations. Certainly, population size and the proximity of animals within such large roosts presents an opportunity for both inter- and intra-species transmission between individuals within a single roost [36]. Both bite and non-bite routes have been suggested as viable mechanisms of virus infection that can be achieved by colonial animals living in high densities [37]. A different picture is present for European microbats that support EBLV-1, EBLV-2 and BBLV maintenance. For all three of these lyssaviruses, the primary reservoirs form comparatively small roosts ranging from 10 to 300 individuals [38,39]. Serological assessments of European bat populations have determined seroprevalence estimates of between 1% and 4% [40,41]. However, the legal framework that protects European bat species has largely precluded meaningful assessment of virus infection in insectivorous European bat species. Where experiments have been undertaken, sample sizes have been small, and results have been difficult to interpret [42,43,44]. Most often, only intracranial inoculation has led to the development of disease reproducibly, with inoculation by various peripheral routes often failing as animals may fail to develop clinical disease and/or seroconvert following inoculation [42,43,44]. Of the few themes that appear conserved between experimental and naturally infected animals, it is clear that the virus can be detected in multiple organs during late stage disease, presumably reaching sites distant to the brain following centrifugal spread [44]. Alongside this, stimulation of an innate immune response to infection also seems comparable in both natural and experimental infection suggesting that regardless of exposure route, innate cell markers are activated in a conserved manner [45]. This latter feature corroborates with molecular experimentation investigating mechanisms of immune avoidance by the lyssaviruses as a genus [46,47,48,49]. 

From the perspective of transmission, the detection of EBLV-2 in oral swab material from both naturally and experimentally infected bats has suggested that EBLVs may be transmitted through biting or scratching [44,50]. However, attempts to detect virus in fecal material or other bodily fluids have largely been unsuccessful [51]. Despite difficulties in detecting virus in swab material and transmission dynamics between bats being undefined, recent studies have suggested that for infection to occur, as long as viable virus reaches a neuron, infection can proceed and clinical disease develops. The minimal infectious dose for these viruses must also be linked strongly to virus maintenance and transmission although again there is little data to indicate a minimal dose. A recent murine study has suggested that for the development of clinical disease in mice, as few as four infectious virus particles may be sufficient when inoculating via a peripheral route [52]. Although experimental, these data suggested that the infectious dose for the lyssaviruses is low and certainly some human infections, especially those involving transmission from insectivorous bats support this finding. A well-documented example of this was a human fatality associated with DUVV infection. Here a woman developed rabies 28-days after having had a small insectivorous bat fly into her face whilst on holiday in Kenya. In this case the individual involved reported no scratch following the incident with the impact not drawing blood. However, despite this the effect of a bat flying into her face permitted the transmission of enough virus to cause productive infection and death [53,54]. In contrast, levels of viable RABV excreted in the saliva of terrestrial mammals have been reported as being significantly greater, possibly indicating that high infectivity is unlikely to reflect an evolutionary adaptation to maximise transmission [55,56,57]. It is generally accepted that the effect of inoculum dose on pathogenicity for the lyssaviruses is complex and that further experimental studies are required to understand infection and transmission dynamics. 

## 4. Incubation Period

Linked closely to the transmitted dose is the incubation period following infection, prior to the onset of clinical disease. This period is highly likely to be a factor of infecting dose and the cell types encountered upon infection. Certainly, the ability of virus to infect different cell types to establish a productive infection is likely to be intrinsically linked to the duration of the pre-clinical period. However, there have been numerous reports that suggest that somehow, even following transmission, the establishment of productive infection may take unusually long periods of time with some cross species transmission events from animals into humans causing clinical disease after extensive incubation periods [58,59]. For experimental studies, where groups of animals have been inoculated with a constant dose of virus via different routes, the pre-clinical period has been reported to vary. For example, the experimental inoculation of Serotine bats with a high dose of EBLV-1 led to clinical disease after 7 to 13 days following intracranial inoculation, 17 to 18 days following subcutaneous inoculation and as long as 26 days following intramuscular inoculation [43]. For EBLV-2, experimental subdermal infection of a Daubenton’s bat with a high inoculation dose led to a pre-clinical period of 32 days [44]. 

From the perspective of natural infection a single report exists documenting the development of disease in a Daubenton’s bat following 9 months in isolated captivity. The trigger for virus activation in this instance is difficult to conclude as the bat was held in a rehabilitation centre prior to developing disease [60]. For bats from temperate climates, such long incubation periods could be seen as beneficial to the virus as it may enable maintenance of the virus during periods of hibernation. Similarly, for migratory bats such incubation periods may aid maintenance of virus during periods where contact rates between animals are minimal, ensuring that the virus is present when animals come together again at roost sites. Elements such as these may allow persistence within metapopulations by increasing the chances of an infected animal being introduced into a population as studied for other viruses [61,62,63,64]. These factors also serve to prevent the removal of an infected individual from a colony and thus low pathogenicity and long incubation periods favour virus maintenance within a population [61].

## 5. Serological Profiles

A number of reports have detailed the presence of lyssavirus neutralising antibodies within healthy bats and the relevance of these findings with respect to the maintenance, transmission and pathogenicity of different viruses has been discussed [65]. Lyssavirus infections have been retrospectively detected through the assessment of bat sera from several countries in Southeast Asia, including the Philippines, Cambodia, Thailand and Bangladesh [66,67,68,69] with rabies virus neutralising antibodies having also been detected in bats from southern China [70]. Despite this, bats of the Old World have never been shown to carry rabies virus, either by antigen detection or virus solation. The significance of these antibody responses remains unclear, especially when levels of neutralising antibodies have been described in both highly colonial insectivorous and frugivorous bats as well as species that exist within comparatively small roost structures. Again, knowledge gaps in bat ecology and population dynamics preclude meaningful assessment of seropositivity within populations and further studies are required. 

Interestingly, experimental attempts to assess serological profiles following virus exposure have also been inconclusive [24,71]. Following multiple experimental challenges with lyssaviruses in both chiropteran and murine experimental models, serological positivity did not necessarily follow infection and, in one study where it did, only 35% of animals that received multiple experimental exposures to titred virus developed a detectable neutralising antibody response [71]. Contrary to what was expected, re-infection did not result in increased numbers of animals developing disease or an anamnestic antibody response being generated [71]. Similarly, murine studies with European lyssaviruses (EBLV-1 and -2) also concluded that multiple exposure did not necessarily lead to either clinical disease or a measurable serological response to infection [52]. These studies have highlighted important questions regarding lyssavirus maintenance and transmission. For example, in the case of lyssavirus infection, how often can animals be exposed to virus without developing either a productive infection leading to clinical disease or a detectable serological response? Another interesting finding from controlled experimentation is the observation that bats deemed to be seronegative have subsequently developed a potent neutralising response following experimental infection whilst other seronegative or bats with low antibody titres have maintained their low antibody titre post infection and survived, regardless of the infective dose administered [72]. Such responses may indicate the presence of antibodies in some bats that may be below the threshold for detection using the current tests. Ultimately, the mechanisms behind the generation of neutralising antibodies following infection is largely unknown and further studies are required to determine the innate response to infection. 

Regardless, the detection of healthy seropositive bats in different roost populations suggests that bats can be infected and clear infection, with no cost to the bat. There has also been a single study investigating antibodies in unvaccinated individuals in an Amazonian human population. This study suggested that humans were being exposed to rabies virus, most likely through transmission from vampire bats, and were clearing infection and seroconverting in the absence of clinical disease [73]. Further investigations into such findings are warranted. In contrast, abortive infection has been hypothesised in animals where experimental infection with viable inocula has led to a complete lack of serological response or disease suggestive of a productive infection [74]. Such contrasting data supports further studies to determine the factors associated with virus clearance, seroconversion and the role for poorly studied immune factors. In particular, a more extensive assessment of the role of the bat immune system and innate effectors in virus clearance is required [75,76].

## 6. Novel Lyssavirus Detection

In the past decade, the increased interest in bats as a host for viruses in general has led to them being studied at an unprecedented level and has resulted in as many new lyssavirus species being detected in the past ten years as had been detected in the previous fifty years. Indeed, the detection of viruses generally has increased exponentially with the ability to generate full sequence data from samples obtained in the field. There are currently 14 lyssaviruses, each assigned as defined species within the lyssavirus genus [77]. For the lyssaviruses, the detection of novel species has generally come in the form of single isolates of viruses from bat species across the Old World. One recent exception to this is the discovery of Ikoma Lyssavirus in a rabid African civet in 2009 [78]. In this instance an African Civet displaying clinical disease consistent with rabies infection was terminated after it aggressively attacked a child within the Ikoma ward of the Serengeti National Park (SNP), Tanzania. The sample was investigated alongside samples from canines that surround the SNP, an area where human habitation and dog ownership are forbidden for habitat and wildlife conservation purposes. The location of the incident was of interest as it was central to the SNP, and some distance from human settlements peripheral to the park. Genetic analysis of the Civet brain revealed that the causative agent of the disease the animal was exhibiting was another lyssavirus, that was genetically highly divergent from all local canine RABV isolates [78,79]. Further genetic assessment, virus isolation and characterisation concluded that this isolate was a new, highly divergent lyssavirus that was subsequently named Ikoma Lyssavirus after the Ikoma ward where the Civet had been terminated [78,79]. Importantly, vaccination challenge experimentation has shown that the current rabies vaccines are unable to protect against this virus due to the high level of antigenic divergence present within the virus glycoprotein [80]. As such the potential threat of this virus to both public and animal health in the area, should an individual be exposed, is enhanced as current vaccines and prophylaxis are unable to protect against the virus. Despite being found as the infecting agent of a terrestrial carnivore, the solitary nature of the rabid host may indicate that IKOV is a bat virus that has infected a terrestrial species. Indeed, African Civets are nocturnal scavenging carnivores and such it may be that the animal was infected following interaction with or ingestion of a grounded bat. 

Whilst the majority of novel lyssaviruses exist as single isolates, one novel lyssavirus has been detected on three occasions within a short time period, an occurrence quite unusual for lyssavirus detection. Bokeloh bat lyssavirus was originally discovered in Germany in 2010 in a Natterer’s bat (*Myotis natterii*) and it was initially assumed that this isolation would represent a further novel single isolate of a lyssavirus [81]. The detection of EBLV-1 and -2 in German and French bat populations has meant that these populations are some of the most intensively studied bat populations in Europe. However, despite several years of active and passive surveillance across Germany and France, within three years three isolations of BBLV have been made, all from Natterer’s bats [82,83,84,85]. With these isolations in mind, attempts at retrospective detection of BBLV have been made across several European countries but no further evidence for the virus has been reported. Where this novel lyssavirus has come from and why within the space of a few years, there have been three isolates of this virus remains puzzling.

It is likely that further novel lyssaviruses will continue to be discovered. Another interesting lyssavirus detection remains to be fully characterised: that of Lleida Bat Lyssavirus (LLEBV) RNA in brain material of a Bent Winged Bat (*Miniopterus* sp.) in Spain [86]. Although few, these genetic data reported for this potentially novel lyssavirus suggests that LLEBV may represent the most genetically divergent lyssavirus discovered to date [86]. Unfortunately, virus isolation with material from the infected bat remains elusive and as such further characterisation is not currently possible. However, the mere suggestion that further novel lyssaviruses exist in bat populations in the EU is of significance to public health. 

**Figure 3:**
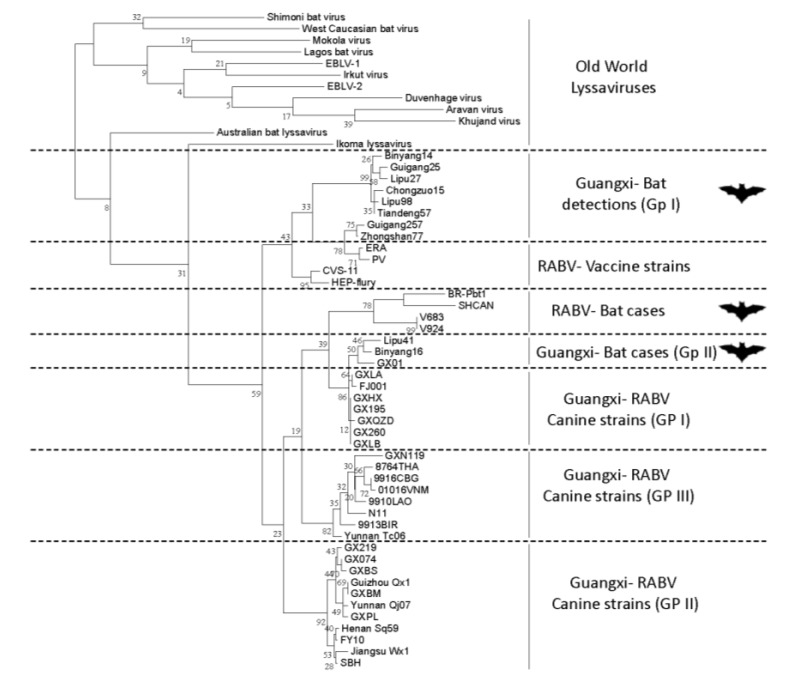
Phylogenetic analysis of lyssavirus sequence data derived from bats in China with rabies and other lyssaviruses. A comparison of 260 nucleotides of the nucleocapsid gene was used to generate the alignment using a maximum-likelihood neighbour-joining analysis.

Whilst the detection of RABV in bats of the Old World has not been confirmed, recent studies have suggested the presence of further lyssaviruses in bat species within China. A number of sequences related to the lyssavirus genus were described from a variety of different bat species [87] (Figure 3). This study reported a low detection rate of lyssavirus nucleic acid in brain material from bats collected over a five year period with only 2.86% (*n =* 85) of samples tested (*n =* 2969) generating a positive amplicon for the N gene [88]. Again, attempts to generate live virus from samples collected proved unsuccessful but efforts continue to further characterise this material [88]. Related to this, the recent detection of Irkut virus in China from a Tube nosed bat (*Murina leucogaster*) is also of interest and in this instance generation of genetic data and virus isolation was successful [89,90].

## 7. Conclusions

Clearly there is something about lyssavirus infection of bats that differs from the relationship that is seen between other pathogens and different bat species [22,25]. Whilst bats are considered the natural reservoir for lyssaviruses, infection with these viruses, with an outcome of clinical disease can and does occur. In North America, where rabies virus has been eliminated from domestic animals, bat rabies continues to constitute a significant zoonotic threat [30,31]. However, when assessing the presence of lyssaviruses within bat roosts, a high level of specific neutralizing antibodies have been reported, suggesting that bats have been exposed to virus but have cleared infection [38,40,41]. The significance of such antibody responses remains unknown although it is of interest that experimental studies with peripheral inoculation of bats with lyssaviruses have often failed to stimulate a neutralizing antibody response. In either case, what has happened to the infecting virus is of great interest. For natural infection it is plausible that an as yet undefined immunological process has prevented the establishment of a productive infection whilst existing virus antigen has been processed as the stimulant for the production of neutralizing antibodies. However, in cases of experimental inoculation, where a high dose of virus has been inoculated peripherally, the lack of disease development and serological response are hard to explain [42,43,44]. Certainly, both experimental studies and a greater understanding of the immunological competence of bats are required to further understand pathogenesis in bats. 

Alongside these features of disease the long incubation period sometimes associated with infection is also of great interest. Knowledge surrounding the duration of pre-clinical incubation periods is largely established from human cases where individuals have been unknowingly infected and have developed fatal disease long after any documented potential exposure [58]. Incubation periods in naturally infected dogs are rarely established although it is generally accepted that if a dog is infected then clinical disease will develop within several weeks. However rare cases of long incubation periods have been reported for bats as described for the natural infection of a Daubenton’s bat that had a minimal incubation period of nine months [60]. The question is what is happening to the virus during these long periods of incubation? Is the duration of incubation period intrinsically linked to the host cell that the virus initially encounters? Does the ability of virus to efficiently replicate and spread between cells rely on host molecules that may act as molecular chaperones for different stages of the viral life cycle? All of these features of lyssavirus replication require investigation to enable a better understanding of infection. 

In conclusion, whilst the risk of lyssaviruses to both the human and animal population from bats remains low, the invariably fatal outcome of clinical disease means that suspected exposures should be followed up appropriately [1]. Cross species transmission events from bats to terrestrial species have been reported (e.g., sheep, horses, cats* etc*. -comprehensively reviewed in [25]), although these events are considered to be transmission to dead end hosts and onward transmission does not appear to occur readily. However, caution is advised as lyssaviruses have been reported as being able to cross the species barrier and establish onward transmission in the newly infected species. Thus there remains the potential for cross species transmission of the more divergent lyssaviruses as has been seen for rabies viruses that have crossed from bat species into terrestrial carnivore species with the establishment of a terrestrial transmission chain [90,91]. This, hypothetically rare, occurrence has been described on two occasions where bat variants of rabies have been discovered in terrestrial species including skunks [90] and red foxes [91]. If such a species jump were to occur again by any of the divergent lyssaviruses and either domestic or wild terrestrial carnivores were able to maintain a transmission chain then a much greater threat to public health could potentially emerge. 
